# Setting Plasma Immersion Ion Implantation of Ar^+^ Parameters towards Electroforming-Free and Self-Compliance HfO_2_-Based Memristive Structures

**DOI:** 10.3390/nano14100831

**Published:** 2024-05-09

**Authors:** Olga Permiakova, Sergey Pankratov, Alexandr Isaev, Andrew Miakonkikh, Yuri Chesnokov, Andrey Lomov, Alexander Rogozhin

**Affiliations:** 1 Valiev Institute of Physics and Technology of RAS, 36/1 Nakhimovsky av., Moscow 117218, Russia; pankratov.sa18@physics.msu.ru (S.P.); miakonkikh@ftian.ru (A.M.); lomov@ftian.ru (A.L.); 2 National Research Centre “Kurchatov Institute”, 1 Akademika Kurchatova Sq., Moscow 123182, Russia; chessyura@yandex.ru

**Keywords:** hafnium oxide, argon ions, nanostructured materials, oxide materials, vacancy formation, ion impact

## Abstract

Memristive structures are among the most promising options to be components of neuromorphic devices. However, the formation of HfO_2_-based devices in crossbar arrays requires considerable time since electroforming is a single stochastic operation. In this study, we investigate how Ar^+^ plasma immersion ion implantation (PI) affects the Pt/HfO_2_ (4 nm)/${\mathrm{HfO}}_{X}N_{Y}$ (3 nm)/TaN electroforming voltage. The advantage of PI is the simultaneous and uniform processing of the entire wafer. It is thought that Ar^+^ implantation causes defects to the oxide matrix, with the majority of the oxygen anions being shifted in the direction of the TaN electrode. We demonstrate that it is feasible to reduce the electroforming voltages from 7.1 V to values less than 3 V by carefully selecting the implantation energy. A considerable decrease in the electroforming voltage was achievable at an implantation energy that provided the dispersion of recoils over the whole thickness of the oxide without significantly affecting the ${\mathrm{HfO}}_{X}N_{Y}$/TaN interface. At the same time, Ar^+^ PI at higher and lower energies did not produce the same significant decrease in the electroforming voltage. It is also possible to obtain self-compliance of current in the structure during electroforming after PI with energy less than 2 keV.

## 1. Introduction

The current stage of artificial intelligence development is based on advances in algorithms. Implementation of artificial intelligence using the von Neumann architecture results in significant power consumption due to the constant data transfer between computing and storage units. Using in-memory neuromorphic [1] devices may lead to an increase in power efficiency. Memristive devices are one of the best candidates for neuromorphic systems due to their low power consumption and nonvolatility [2,3]. The memristor array can do matrix–vector multiplication via Kirchoff’s laws [4], significantly increasing the speed and power efficiency of AI technology. Additionally, memristive devices often have a simple metal–insulator–metal structure. Additionally, memristors based on binary oxides are compatible with CMOS technology [3]. However, several difficulties arise during crossbar operation: the sneak-path issue [5], device-to-device variability, and the need for electroforming. One approach to avoid sneak-path issues is complementary resistive switching (RS), which has been demonstrated for HfO_2_-based metal–insulator–metal devices [6]. Despite device-to-device variability, RS parameters of structure are often provided for individual devices, such as endurances higher than $10^{10}$ switching cycles [7]. Device-to-device variability of resistive switching characteristics is connected to the random nature of electroforming [8]. Additionally, electroforming is an individual process, which significantly limits the crossbar size due to its complexity and since formation time linearly grows with the crossbar size.

Typical RS in HfO_2_-based memristors takes place due to the formation and reduction of conductive filament [9,10,11]. The initial conductive filament is generated by electroforming, during which voltage is applied to the device until dielectric breakdown (BD). So electroforming requires larger voltages than what are needed during device operation. For this article, we assume that electroforming occurs according to the recently presented charge injection model of the dielectric breakdown [12].

Several methods were proposed to induce electroforming-free behaviour in memristive structures. The most common one is optimization of structure deposition [13,14]: in particular, deposition of non-stoichiometric films [15,16,17]. For example, the value of the forming voltage was drastically lowered to the values of switching voltages in non-stoichiometric hafnium oxide with thicknesses up to 50 nm [15]. However, there is no evidence for an increase in the resistance ratio for structures with a thicker oxide. Additionally, oxygen deficiency in the switching oxide could lead to early endurance degradation in memristive devices [18]. Thoughtful defect engineering with texture transfer via molecular beam epitaxy has allowed researchers to reduce the forming voltage below 2 V in 10 nm hafnium oxide [13]. Another method includes ion post-processing, which was shown to lower electroforming voltages, as well as the variability of RS parameters in valence change memory devices, which are discussed further in the text. Other methods of induced electroforming-free behaviour in memristive devices include photo-assisted methods [19], oxide doping [20], and thermal treatment [21]. Photo-assisted electroforming [19] of ${\mathrm{MAPbI}}_{3}$-based structure allows improved reliability of memristive devices by reducing the overshoot current during switching processes. However, the photo-assisted method can only be used for light-sensitive materials.

Previous studies utilized plasma treatment and ion implantation (irradiation) on memristive structures to improve their RS characteristics [22,23,24,25,26,27]. Two types of ions are usually used: (i) inert gas ions—to generate a displacement cascade with impact-induced structural damage, which serves as an origin for conductive filament and (ii) chemically active ions—to alter the activation energies of chemical reactions or change the stoichiometry of the structure. For example, Ar plasma treatment of hafnium oxide prior to Ti-top electrode deposition made electroforming unnecessary and improved endurance, retention, switching speed, and cycle-to-cycle variability of RS characteristics [22]. As such treatment leads to an increase in the surface roughness and a thicker ${\mathrm{TiO}}_{X}$ interface layer at the Ti/HfO_2_ (9 nm), the improved performance was attributed to the growth of compact filaments. Ar plasma treatment with of ${\mathrm{TaO}}_{X}$ (2 nm)/InGaZnO (4 nm) lowered the variability of RS characteristics [23]. Ar^+^ implantation with an energy of 17 keV and a fluence range of $6.90\times 10^{14}-1.73\times 10^{16}$ ${\mathrm{cm}}^{-2}$ enables electroforming in 30 nm ${\mathrm{SiO}}_{X}$, which was not possible without implantation [24]. Xe^+^ irradiation with an energy of 5 keV and a fluence range of $1\times 10^{11}-1.1\times 10^{13}$ ${\mathrm{cm}}^{-2}$ on a ${\mathrm{SiO}}_{2}$ (40 nm) surface increased the resistance ratio and decreased the electroforming voltage variation [25]. Gd^+^ [26] and ${\mathrm{Ti}}^{+}$ [27] ion implantation provided the localization of oxygen vacancies and reduced the variability of RS characteristics.

The effect of implantation on the electroforming process has received little attention, despite the high interest in the effect of inert gas ion surface treatment and the belief that the random nature of electroforming leads to device-to-device variability [8]. Even though electroforming data are analysed, this is based on average values corresponding to Gaussian distributions and voltage ranges for a small set of measured structures. This complicates the comparison of experimental and calculated data.

Dielectric BD is usually characterized by time-to-BD obtained by constant-voltage stress (CVS) [28]. In this case, time-to-BD is described by the Weibull distribution in accordance with percolation theory. Subsequently, it is possible to estimate the critical density of defects and the area of the degraded spots [29] and compare experimental data with calculated data based on a model of oxide degradation [30]. It has been shown that fast ramp-voltage stress (RVS) measurements can be converted to slow CVS measurements. RVS suits well for ${\mathrm{HfO}}_{2}$ characterization, as it has been proposed for high-k dielectrics due to the large time-to-BD variance [31]. Additionally, extreme value analysis has provided a good description of RS voltages as well as the distribution of resistance in RS states between switching cycles [32,33].

In this article, we have investigated the influence of plasma-immersion ion implantation (PI) on electroforming parameters in ${\mathrm{HfO}}_{2}$-based structures. To study the effect of PI directly on the insulator itself, we used ${\mathrm{Ar}}^{+}$ PI before the deposition of the Pt electrode. In order to select the optimal implantation parameters, we studied the influence of processing on the electroforming. The RVS method was used for fast analysis of electroforming voltages for a large set of devices. To describe the BD voltages in the insulator, we used the Weibull distribution. We demonstrated that the proper choice of implantation parameters not only provided a decrease in forming voltage and its variability but also led to current self-compliance even for electroforming. Transmission electron microscopy (TEM) and scanning transmission electron microscope (STEM) measurements were used to analyse the investigated structure, including the energy-dispersive X-ray spectroscopy (EDS) line scan profile, which showed the importance of the displacement of hafnium atoms on the shape of the electroforming voltage distribution. We also showed that the roughness increase near the Pt/${\mathrm{HfO}}_{2}$ interface is hardly a reason for electroforming-free behaviour: it was just a side effect of implantation. Additionally, we showed that the BD voltage distribution of implanted devices changes if subjected to the air for a few hours. As a result, it is not obvious if an ex situ analysis would be valid in our case.

## 2. Materials and Methods

In this article, we investigated the effect of PI parameters on BD voltage in a ${\mathrm{HfO}}_{2}$-based structure. A ${\mathrm{HfO}}_{2}$ (4 nm)/${\mathrm{HfO}}_{X}$$N_{Y}$ (3 nm)/TaN (5 nm) structure was formed by atomic layer deposition (FlexAL, Oxford Instruments Plasma Technology, Yatton, Bristol, UK) on top of TiN (100 nm)/Si. A detailed description of the recipes in use can be found elsewhere [6]. Top electrodes (Pt) with a thickness of 100 nm were deposited via magnetron sputtering (Balzers SCD 050) through a shadow mask. The area of the electrodes was equal to 0.036 ${\mathrm{mm}}^{2}$.

${\mathrm{Ar}}^{+}$ implantation was performed via plasma immersion ion implantation (PI) by a custom tool with a 13.56 MHz inductively coupled plasma source. The inlet RF power was 600 W and was supplied via an impedance matching device. The chamber base vacuum level was $2\times 10^{-5}$ mbar. PI was conducted at room temperature with a process pressure of 5.0±0.5 mbar set with a 50 sccm flow of Ar gas. Implantation was performed by applying 10-μs rectangular pulses of negative accelerating potential (1 kV to 4 kV) with a frequency of 1 kHz (duty cycle is 0.01). Implantation fluence was calculated using:

(1) $$fluence=\frac{j_{0}\Delta t}{e},$$

where $j_{0}$ is ion current density averaged with measured current impulse value and duty cycle, Δt is total implantation time, and *e*—electron charge. A sample was placed in a steel or aluminium 150 mm chuck.

The microstructure of the devices was also investigated. The cross-sectional specimens were prepared by a focused ${\mathrm{Ga}}^{+}$ ion beam (FIB) in a Versa 3D (ThermoFisher, Waltham, MA, USA) dual beam microscope. Transmission electron microscopy (TEM) and scanning transmission electron microscopy (STEM) studies were performed employing a TEM/STEM Osiris microscope (ThermoFisher Scientific, Waltham, MA, USA) at an accelerating voltage of 200 keV. The microscope was equipped with a high-angle annular dark-field detector (Fischione, Cleveland, OH, USA) and a Super-X energy dispersive X-ray spectrometer (Bruker, Bremen, Germany). Additionally, to check the crystallinity of the structure, grazing incidence X-ray diffraction (GIXRD) measurements with a sliding angle of 0.5° were performed via a Rigaku diffractometer (SmartLab, Tokyo, Japan).

Electroforming of the devices was performed using a Keithley-4200 SCS, Cleveland, OR, USA with ramp-voltage stress (RVS) [31]. The device was subjected to a step-increasing negative voltage with a voltage step of −0.1 V from 0.0 V and a time step of 1.0 s. An external current compliance limit of 0.1 mA was set during electroforming. As such, electroforming led to follow-up RS in the reference structure. When the current through the structure achieved current compliance, electroforming was stopped. An abrupt current increase pressed the electroforming, so the breakdown voltage was defined as the voltage before a sudden increase in current (Figure S1). To prevent significant overshoot, a 18 kOhm resistor was connected serially to the device.

I-V characteristics were measured with a trans-impedance amplifier TLC2201 and QMBox setup, which included a DAC (QMS45), which was used as a pulse generator, and an 18-bit ADC (QMS17), which was used as an oscilloscope. No external current compliance limits were used during the I-V measurements. During all measurements, voltage was applied to the top electrodes, while the bottom one was grounded.

Surface roughness was characterised by atomic force microscopy (AFM, SMM-2000) with a CSG01 contact probe before the deposition of a Pt electrode. Images of the surface roughness obtained via AFM measurements are provided in the Supplementary Materials.

### Data Analysis

Defect generation is a stochastic process that shapes the electroforming process; hence, the appropriate distribution is required to characterise it. For CVS measurements, the time-to-BD distribution usually corresponds to the bimodal Weibull distribution, for which the cumulative density function (cdf) is given by:

(2) $$cdf=1-exp{\left(-\frac{t}{t_{63}}\right)}^{\beta },$$

where *t*—time-to-BD, $t_{63}$ is the scale factor at 63.2%, and β is called Weibull slope or shape factor. Usually, BD data are presented in the form of a Weibull plot as $W\left(t\right)=ln\left(-ln\left(1-cdf\right)\right)=\beta \left(ln\left(t\right)-ln\left(t_{63}\right)\right)$. Typically, shape factors of bimodal distribution are considered to represent intrinsic and extrinsic BDs. An extrinsic BD has a flatter distribution and is caused by defects induced during the structure’s formation before CVS, whereas an intrinsic BD has a steeper distribution and is caused by the CVS procedure. The BD voltage of hafnium oxide subjected to RVS can be described in the same way, but it scaled by a factor [31]. The time-to-BD of ${\mathrm{HfO}}_{2}$ to the BD voltage is approximated with a power-law [28]. Similar conclusions can be drawn from RVS measurements, but due to a lack of information on the voltage acceleration model itself, it is difficult to determine the parameters of the dielectric without translating RVS to CVS data [28].

## 3. Results

**Figure 1:**
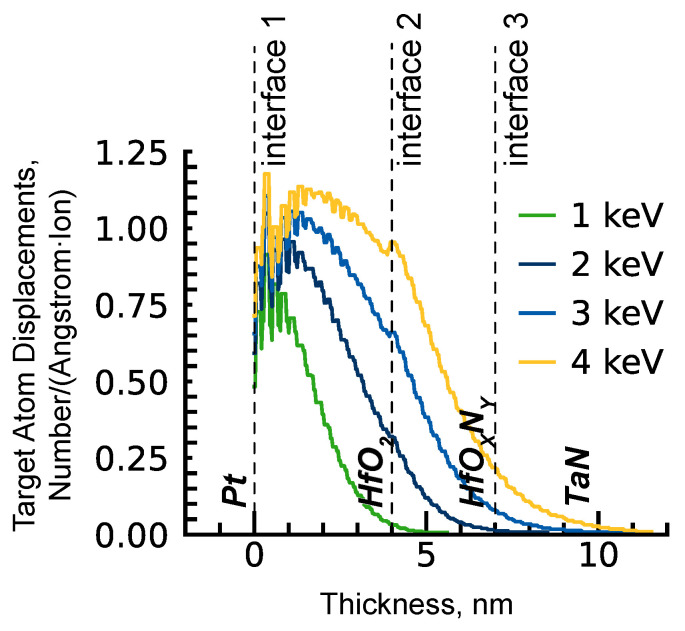
SRIM simulation of displacements induced by ${\mathrm{Ar}}^{+}$ plasma immersion ion implantation in ${\mathrm{HfO}}_{2}$/${\mathrm{HfO}}_{X}$$N_{Y}$/TaN structure.

We used the implantation parameters given in Table 1. The PI energy range was chosen based on an SRIM simulation (Figure 1) [34], according to which:

- 1 keV—recoil distribution extends along the entire thickness of ${\mathrm{HfO}}_{2}$ and slightly influences interface 2;
- 2 keV—recoil distribution extends along the entire thickness of the insulator and does not influence interface 3;
- 3 keV—recoil distribution extends along the entire thickness of the insulator and slightly influences interface 3;
- 4 keV—recoil distribution extends along the entire thickness of the insulator and significantly influences interface 3.

In order to obtain a forming-free device, the initial vacancy concentration needed to be equal to the pre-BD vacancy concentration. So the PI ion fluence ($6.0\times 10^{15}$ ${\mathrm{cm}}^{-2}$ for 3 keV) was estimated to meet an oxygen vacancy concentration of approximately 10% in ${\mathrm{HfO}}_{2}$ [30]. Additionally, it is considered that only 1% of the calculated recoil numbers computed in SRIM would provide vacancy due to the self-annealing effect, as no thermal effects are allowed in SRIM. To describe only the effect of energy, fluences were recalculated to give an equal number of peak oxygen recoils in accordance with SRIM results. A higher fluence of $2.0\times 10^{16}$ ${\mathrm{cm}}^{-2}$ for the same PI energy was also realised (Table 1).

**Figure 2:**
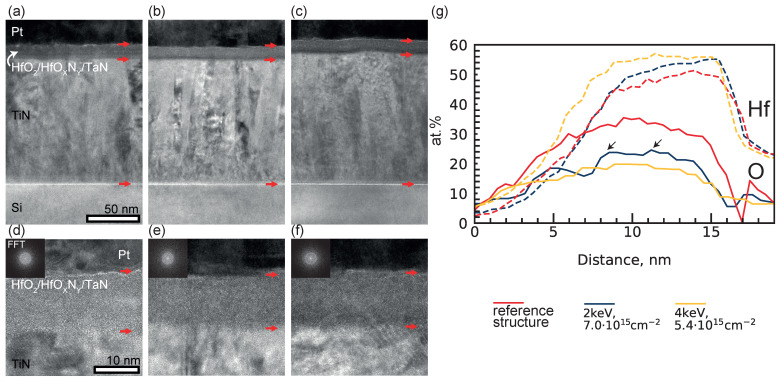
Cross-sectional transmission electron microscope (TEM) images of Pt/${\mathrm{HfO}}_{2}$/${\mathrm{HfO}}_{X}$$N_{Y}$/TaN memristive structures: (**a**,**d**) Cross-sectional TEM of reference structure, (**b**,**e**) structure after ${\mathrm{Ar}}^{+}$ PI with energy 2 keV and fluence $7.0\times 10^{15}$ ${\mathrm{cm}}^{-2}$, (**c**,**f**) structure after ${\mathrm{Ar}}^{+}$ PI with energy 4 keV and fluence $5.4\times 10^{15}$ ${\mathrm{cm}}^{-2}$. Red arrows indicate the boundaries of the amorphous layer. Inset: typical FFT diffraction pattern of the amorphous region ${\mathrm{HfO}}_{2}$/${\mathrm{HfO}}_{X}$$N_{Y}$/TaN. (**g**) EDS line scan profiles of initial structure (red lines) and structures after ${\mathrm{Ar}}^{+}$ PI with energies of 2 keV (blue lines) and 4 keV (yellow lines) for Hf (dotted lines) and O (solid lines). Black arrows indicate features associated with the ${\mathrm{HfO}}_{X}$$N_{Y}$ interfaces.

We investigated the structure’s morphology via transmission electron microscopy (TEM) (Figure 2). Figure 2a shows that the TiN layer has a “column” structure with a column width of 20–40 nm. The columnar structure results in a rough TiN surface, due to which the thickness of the layer varies from 123 to 127 nm. A high-resolution TEM image is shown in Figure 2d. Analysis of the interplanar distances ($d_{<111>}$= 2.4 Å, $d_{<200>}$= 2.1 Å) shows that TiN has a cubic face-centred crystal lattice (unit cell parameter a = 4.24 Å). The amorphous layer on the surface of TiN has a thickness of 14–15 nm. The FTT spectrum in the inset shows that the layer is completely amorphous (Figure 2d, inset); detailed analysis shows a complete absence of nanocrystalline inclusions in it. No crystalline hafnia was observed during GIXRD measurements either (Figure S3). High-resolution TEM images do not allow us to determine the location of layer boundaries in the amorphous ${\mathrm{HfO}}_{2}$/${\mathrm{HfO}}_{X}$$N_{Y}$/TaN ALD structure (Figure 2d). A layer of crystalline Pt with a cubic face-centred lattice (unit cell parameter a = 3.85 Å, interplanar distance $d_{<111>}$= 2.2 Å) was located on top of this layer. TEM images of this multilayer structure show no significant changes after implantation with ${\mathrm{Ar}}^{+}$ ions, as shown in Figure 2a–f. The only noticeable change after implantation is the darkening of the location, which corresponds to the TaN layer near the TaN/TiN interface. After implantation, the amorphous ${\mathrm{HfO}}_{2}$/${\mathrm{HfO}}_{X}$$N_{Y}$/TaN structures remained amorphous without nanocrystalline inclusions.

To investigate the change to the ${\mathrm{HfO}}_{2}$/${\mathrm{HfO}}_{X}$$N_{Y}$/TaN structure in more detail, HAADF STEM images were acquired (Figure S4a). The HAADF STEM image shows areas of reduced intensity in the centre of the ${\mathrm{HfO}}_{2}$/${\mathrm{HfO}}_{X}$$N_{Y}$/TaN layer, which roughly corresponds to the location of the ${\mathrm{HfO}}_{X}$$N_{Y}$ layer. The size of the regions with this contrast is about 2 nm. Such features were observed, for example, in the HfN layer in [35]. The reduced density and, hence, intensity in such regions were explained by nanoporosity. However, in the corresponding work, Ar plasma was used as a precursor, and in this work, ${\mathrm{Ar}}^{+}$ ions are used for post-processing of ALD-grown films. HAADF STEM images (Figure S4b,c) of the multilayer structure after ${\mathrm{Ar}}^{+}$ ion implantation show that the regions are less localized and are observed in the upper 9 nm of the ALD structure, which corresponds to the conventional ${\mathrm{HfO}}_{2}$ and ${\mathrm{HfO}}_{X}$$N_{Y}$ layers. The feature sizes are reduced to 1–1.5 nm. Thus, significant mixing of the material between the ${\mathrm{HfO}}_{2}$ and ${\mathrm{HfO}}_{X}$$N_{Y}$ layers happened during ${\mathrm{Ar}}^{+}$ implantation.

However, it is worth considering that the samples may have oxidised significantly in the atmosphere after the preparation of thin lamellae. Thus, elemental EDS mapping shows (Figure 2g) a significant amount of oxygen: more than 6 at% in the whole ${\mathrm{HfO}}_{2}$/${\mathrm{HfO}}_{X}$$N_{Y}$/TaN structure. Thus, EDS analysis may not show the real distribution of elements in the multilayer structure, and complementary methods are required to accurately determine the composition of the layers. Despite this, trends of implantation effects are clearly seen from the EDS line scan profiles for Hf and O (Figure 2g). For oxygen, it seems that its composition decreased equally for different implantation energies. However, unlike implantation with an energy of 4 keV, which simply shifted the oxygen profile to lower composition values, implantation with an energy of 2 keV allowed us to change the shape of the profile itself. After implantation with an energy of 2 keV, two features are clearly visible on the oxygen profile, which are indicated by arrows in Figure 2g. The distance between these features is close to the thickness of ${\mathrm{HfO}}_{X}$$N_{Y}$. For hafnium, it seems that implantation with lower energy (2 keV) preserves the hafnium composition along the thickness of the structure; however, implantation with higher energy broadens the hafnium composition along the structure thickness, thus leading to the mixing of Hf and TaN in the neighbouring layer.

**Figure 3:**
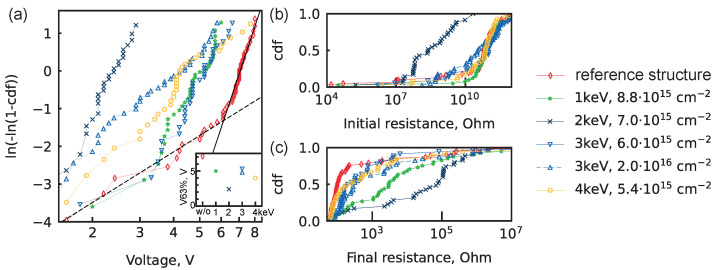
Distributions for Pt/${\mathrm{HfO}}_{2}$/${\mathrm{HfO}}_{X}$$N_{Y}$/TaN structures. (**a**) Weibull plot for breakdown (BD) voltages. Solid black line—intrinsic BD fit; dashed black line—extrinsic BD fit. All fitting lines are shown in Supplementary Figure S2. Parameters of all fitting line are shown in Table 1. Inset: the voltage value at 63.2% cdf versus the implantation energy. (**b**) Cumulative distribution function (cdf) of pre-BD resistance in devices subjected to electroforming. (**c**) The cdf of post-BD resistance in devices subjected to electroforming.

Figure 3a shows the distribution of BD voltages for the structures under investigation (Supplementary Figure S2 shows the plot with potential fitting lines); the fitting line parameters are displayed in Table 1. Figure 3a clearly shows that PI ions have a particular energy (2 keV) that provides the lowest electroforming voltage. Note that one point from the unipolar distributions of BD voltages for energies 1 keV and 3 keV is not taken into account when fitting. The points are inherited from the extrinsic distribution of the BD voltage of the reference structure, and the probability of the occurrence of such events is less than 5% for the implanted structures with the considered processes.

### 3.1. Effect of Fluence Increase

The effect of fluence increase was checked for ${\mathrm{Ar}}^{+}$ ions with energy 3 keV (Figure 3). For PI with lower a fluence of $6.0\times 10^{15}$ ${\mathrm{cm}}^{-2}$, BD voltages were fitted with a unimodal Weibull distribution with a slope of 5.8. A further increase in the ion fluence to $2.0\times 10^{16}$ ${\mathrm{cm}}^{-2}$ led to a bimodal distribution with slopes of 5.9 and 2.5. The transition from an intrinsic to an extrinsic distribution of BD voltages takes place at 5.1 V. Accordingly, there is a transition from a unimodal distribution to a bimodal distribution when a larger ion fluence is used. The larger fluence of ions should result in additional defects in the implanted structure, which gives rise to an extrinsic BD mechanism.

### 3.2. Effect of Energy Increase

Minimal electroforming voltage was obtained for PI with ion energy of 2 keV, whereas PI with lower energy (1 keV) and higher energies (3 keV, 4 keV) had larger BD voltages (Figure 3a, inset). So there is an optimal energy (2 keV) that preserves a narrow BD distribution and reduces the BD voltage to absolute values less than 3 V. ${\mathrm{Ar}}^{+}$ PI with energies of 1 keV and 3 keV leads to a similar distribution of BD voltages. Overall, the BD voltage distributions for 1 keV, 2 keV, and 3 keV are intrinsic, which corresponds to the narrowest distribution of BD voltages possible for this structure. However, ${\mathrm{Ar}}^{+}$ PI with an energy of 4 keV leads to a trimodal distribution of BD voltages, which is not explained in the utilised model and could be related to a substantially destructed interface 3. Additionally, only ${\mathrm{Ar}}^{+}$ PI with optimal energy leads to significantly lower pre-BD resistance (Figure 3b).

### 3.3. Effect of Time before Deposition of Top-Electrode

**Figure 4:**
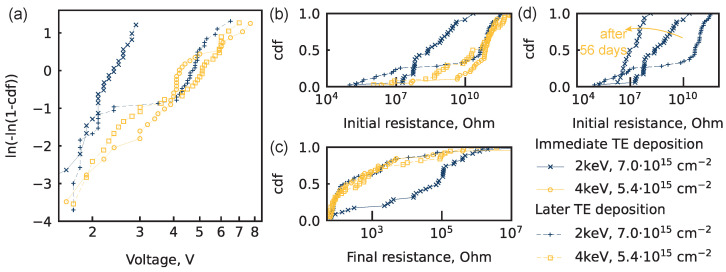
Distributions for Pt/${\mathrm{HfO}}_{2}$/${\mathrm{HfO}}_{X}$$N_{Y}$/TaN structures. (**a**) Weibull plot for breakdown (BD) voltages. All fitting lines are shown in Supplementary Figure S2. Parameters of all fitting lines are shown in Table 1. (**b**) Cumulative distribution function (cdf) of pre-BD resistance in devices subjected to electroforming. (**c**) The cdf of post-BD resistance in devices subjected to electroforming. (**d**) Pre-BD resistance change of the finest structure after 56 days in air.

After ${\mathrm{Ar}}^{+}$ PI, devices were exposed to the air while being transferred to another tool. Top electrodes were formed on some structures immediately after PI and on others the next day. Larger air exposure times led to a change in BD voltage distribution. Similar BD voltage distributions were obtained for devices after PI with energies of 2 keV and 4 keV, which were significantly different if the top electrodes were deposited immediately after implantation (Figure 4a). The resulting distributions are trimodal, although the modes have comparable slopes and are steep (Table 1). Additionally, a significant distribution split into two batches was found for pre-BD resistance after ${\mathrm{Ar}}^{+}$ PI with an energy of 2 keV (Figure 4b). The resistance for 75% of devices became equal to the resistance of the reference structure, and the resistance for 25% of devices decreased compared to that of the structure with immediate electrode fabrication. After 56 days, the initial resistance of the structure with immediate electrode fabrication was measured once more (Figure 4d). The distribution of the pre-BD resistance preserves its form but is slightly shifted to lower values. The effect of such oxidation is not thoroughly explored in this article and is left for future research.

### 3.4. Resistance before and after Breakdown

Additional crucial parameters are the devices’ pre-BD and post-BD resistances (Figure 3b,c), as PI is used for the formation of electroforming-free memristors. The final resistance was expected to be low due to overshoot because of the tool features. Nevertheless, self-compliance was observed for devices with immediate electrode fabrication after ${\mathrm{Ar}}^{+}$ PI with energy less than 2 keV (Figure S1). This is also reproduced in the distribution of post-BD resistances (Figure 3c).

### 3.5. Roughness Analysis

Table 1 provides information about the RMS roughness of the devices under investigation (Figure S5). In comparison to other devices, the reference structure has nearly twice the lower roughness of 0.4 nm. The maximum roughness of 1.1 nm was in the device after PI with energy of 3 keV and fluence of $6.0\times 10^{15}$ ${\mathrm{cm}}^{-2}$. Other structures have similar roughnesses of about 0.7–0.9 nm. In implanted structures with unimodal and bimodal distributions, the structure with the largest roughness has the lowest intercept value. In this study, no further roughness effects were observed.

### 3.6. I-V Characteristics of Structure with Self-Compliance of Current

**Figure 5:**
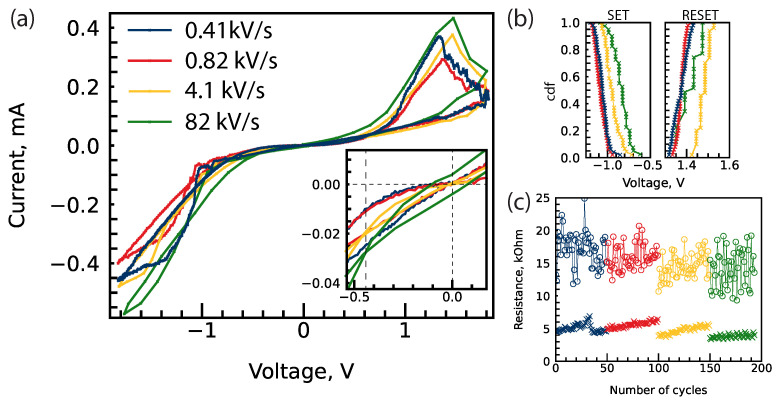
(**a**) I-V characteristics of Pt/${\mathrm{HfO}}_{2}$/${\mathrm{HfO}}_{X}$$N_{Y}$/TiN device at different rates of voltage change. Inset: enlarged section near 0 V stress. (**b**) Cumulative distribution of switching voltages. (**c**) Resistance state at voltage 1.1 V, obtained from (**a**). All resistive switching cycles are presented in Supplementary Figure S6.

I-V characteristics of structures implanted with energy of 2 keV and fluence of $7.0\times 10^{15}$ ${\mathrm{cm}}^{-2}$ were further investigated without current compliance limits (Figure 5a). During I-V measurements, the devices were subjected to voltage sweeps in the range of −1.8 V to 1.8 V at different rates of voltage change from 0.41 kV/s to 82 kV/s. The cumulative distribution of SET and RESET voltages is presented in Figure 5b. The type of I-V characteristics was similar to the nonlinear I-V characteristics described in our previous paper [6]. However, no memory window was observed at negative voltages in the self-compliance regime in the investigated range. The memory window observed before RESET was rather low and did not exceed 3.

Increasing the rate of voltage change increased the current at higher voltages (Figure S6) as well as shrunk the memory window to 2. Despite that, degradation of the low-resistance state was less noticeable for the highest voltage change rate (Figure 5c). Additionally, I-V hysteresis measured at a voltage change rate of 82 kV/s was not pinched at 0 V but rather at −0.44 V (Figure 5a, inset); such behaviour is under investigation.

## 4. Discussion

The distributions of BD voltage in ${\mathrm{Ar}}^{+}$ implanted structures were investigated. They follow the Weibull distribution, similar to the BD voltages of the reference structure. We have found that certain energies (2 keV) permit both a decrease in forming voltages and the preservation of a narrow distribution of BD voltages. According to SRIM simulation, the energy corresponds to the impact-induced damage by ${\mathrm{Ar}}^{+}$ ions along the entire thickness of the active layer, with negligible effect on the bottom electrode interface. In this case, we attribute the decrease in the electroforming voltage to oxide degradation as opposed to effects at the ${\mathrm{HfO}}_{2}$/TiN interface [22]. This is due to the following reasons: (i) we perform inert Pt electrode deposition rather than use a Ti-based electrode; (ii) after lower and higher implantation energies (1 keV and 3 keV), the electroforming voltage at 63.2% cdf is higher (5.0 V and 5.3 V, respectively) compared to the structure after implantation with ion energy of 2 keV (2.4 V).

Changing the implantation energy allows us to not only control the electroforming voltage but also the self-compliance of the device. We have shown that current self-compliance takes place after PI with low energies (1 keV and 2 keV). Previously, the Zhang group attributed self-compliance in ${\mathrm{HfO}}_{2}$-based memristors to the accumulation of oxygen close to the anode interface during SET [36]. This is consistent with our findings, as during PI, oxygen atoms should be dislocated and recoiled deeper into the structure. Based on SRIM results, oxygen remains in the insulator at energies of 1–2 keV because the majority of oxygen atoms are displaced inside hafnium oxide. However, energy increases (3 keV and 4 keV) allow a considerable number of oxygen atoms to recoil further into the next TaN layer, lowering the oxygen concentration inside the insulator. This is also consistent with the data of the EDS line scan profile (Figure 2g), according to which a sharp change in the oxygen profile near the assumed ${\mathrm{HfO}}_{X}$$N_{Y}$/TaN boundary is noticeable for low implantation energy (2 keV). For high implantation energy (4 keV), there were no sharp local changes in the oxygen distribution profile along the structure thickness. Additionally, for an implantation energy of 4 keV, the broadening of the Hf composition profile is noticeable, which may indicate the ejection of Hf into TaN. The SRIM calculation also indicates that a noticeable part of hafnium is pushed into the next TaN layer at ${\mathrm{Ar}}^{+}$ PI with an energy of 4 keV, but this does not occur at an implantation energy of 2 keV (Figure S7). This suggests that the maximum possible implantation energy required to create an electroforming-free structure should not exceed the energy at which there is a significant displacement of the metal atom from the implanted oxide, but further studies are needed to verify this hypothesis.

We further demonstrate, using an appropriate distribution of BD voltages, that excessive implantation fluence may have the undesirable impact of flattening the distribution of BD voltages. At an implantation energy of 3 keV, when ion fluence was raised from $6.0\times 10^{15}$ ${\mathrm{cm}}^{-2}$ to $2.0\times 10^{16}$ ${\mathrm{cm}}^{-2}$, the distribution transitioned from unimodal to bimodal. We attribute the appearance of the second distribution mode to the extensive defect formation in the insulator, similar to BD, due to the defects aroused during structure formation. Consequently, higher implantation fluence leads to the second mechanism of conductive filament formation due to insulator defects, which should increase device-to-device variability. This effect was demonstrated due to the use of the Weibull distribution for the description of the BD voltage distribution.

So we must ask why researchers should form a non-stoichiometric structure with implantation if it can be done just by deposition. Implantation allows for oxygen ions to remain within the insulator. As mentioned previously, oxygen ions pile up near the anode, resulting in device self-compliance during the SET process. Additionally, it was previously shown that oxygen plasma treatment of hafnium oxide leads to an increase in the device’s endurance [18].

## 5. Conclusions

In this article, we investigated the effect of ${\mathrm{Ar}}^{+}$ plasma immersion ion implantation (PI) on the electroforming voltage in a Pt/${\mathrm{HfO}}_{2}$ (4 nm)/${\mathrm{HfO}}_{X}$$N_{Y}$ (3 nm)/TaN structure. We showed that if parameters of implantation are properly chosen, it is possible to significantly reduce electroforming voltage as well as make self-compliance structures. The best results were achieved for implantation when the recoil profile was calculated to span the whole insulator thickness and end right before the interface with an electrode. The EDS line scan profile and SRIM modelling also showed that, in this case, there is no significant displacement of hafnium atoms from the oxide into the next layer. We experimentally demonstrated that there is an upper limit of ion fluence, beyond which the dispersion of electroforming voltage increases. Additionally, we showed that the presence of the structure in air after ${\mathrm{Ar}}^{+}$ PI leads to a change in the form of the BD voltage distribution. So top electrodes have to be immediately deposited after implantation to acquire the best implantation effects.

## Figures and Tables

**Table 1:** Parameters of plasma immersion ion implantation of ${\mathrm{Ar}}^{+}$ ions.

Energy,	Fluence,	Immediate	Roughness ($R_{q}$),	Distribution Analysis *		
keV	${\mathrm{cm}}^{-2}$	Top-Electrode	nm	Type **	Slope	Interception
w/o	w/o		0.4	bimodal	9.3	−18.2
					1.9	−4.8
1	$8.8\times 10^{15}$	+	-	unimodal	5.8	−9.3
2	$7.0\times 10^{15}$	+	-	unimodal	6.5	−5.7
2	$7.0\times 10^{15}$	-	0.8	trimodal	5.8	−8.9
					6.1	−6.0
3	$6.0\times 10^{15}$	+	1.1	unimodal	5.8	−9.7
3	$2.0\times 10^{16}$	+	0.7	bimodal	5.9	−9.2
					2.5	−3.6
4	$5.4\times 10^{15}$	+	-	trimodal	1.8	−2.5
					12.9	−18.5
					2.8	−4.7
4	$5.4\times 10^{15}$	-	0.9	trimodal	4.2	−6.8
					4.2	−5.5

* Approximation for distribution types sorted from highest to lowest voltage ranges. ** Line approximations for distribution types are provided in Supplementary Figure S2.

## Data Availability

Data will be made available on request.

## Supplementary Materials

The following supporting information can be downloaded at: https://www.mdpi.com/article/10.3390/nano14100831/s1, Figure S1. Example of electroforming process of Pt/${\mathrm{HfO}}_{2}$/${\mathrm{HfO}}_{X}$$N_{Y}$/TaN after plasma immersion ion implantation of ${\mathrm{Ar}}^{+}$ with energy 2 keV and fluence $7.0\times 10^{15}$ ${\mathrm{cm}}^{-2}$. Figure S2. FittedWeibull distributions of breakdown voltages for Pt/${\mathrm{HfO}}_{2}$/${\mathrm{HfO}}_{X}$$N_{Y}$/TaN structures subjected to plasma immersion ion implantation of ${\mathrm{Ar}}^{+}$. Figure S3. GIXRD spectra of the ${\mathrm{HfO}}_{2}$/${\mathrm{HfO}}_{X}$$N_{Y}$/TaN structure without implantation. Figure S4. Cross-sectional HAADF-STEM image of the Pt/${\mathrm{HfO}}_{2}$/${\mathrm{HfO}}_{X}$$N_{Y}$/Ta structures and corresponding EDS mapping of N, Ti, O, Pt, Hf and Ta without deconvolution. Figure S5. Images used to estimate surface roughness measured via AFM. The images were processed with a Fourier filter. Figure S6. I-V characteristics of Pt/${\mathrm{HfO}}_{2}$/${\mathrm{HfO}}_{X}$$N_{Y}$/TaN structures subjected to plasma immersion ion implantation of ${\mathrm{Ar}}^{+}$ with energy of 2 keV and fluence of $7.0\times 10^{15}$ ${\mathrm{cm}}^{-2}$. Figure S7. SRIM simulation of Hf atom displacements induced by ${\mathrm{Ar}}^{+}$ plasma immersion ion implantation in ${\mathrm{HfO}}_{2}$/${\mathrm{HfO}}_{X}$$N_{Y}$/TaN structure with energies of 2 keV (a) and of 4 keV (b).

## References

[1] Fetisenkova K.A., Rogozhin A.E. (2023). Neuromorphic Systems: Devices, Architecture, and Algorithms. Russ. Microelectron..

[2] Kumar S., Wang X., Strachan J.P., Yang Y., Lu W.D. (2022). Dynamical memristors for higher-complexity neuromorphic computing. Nat. Rev. Mater..

[3] Dittmann R., Menzel S., Waser R. (2021). Nanoionic memristive phenomena in metal oxides: The valence change mechanism. Adv. Phys..

[4] Li H., Wang S., Zhang X., Wang W., Yang R., Sun Z., Feng W., Lin P., Wang Z., Sun L. (2021). Memristive Crossbar Arrays for Storage and Computing Applications. Adv. Intell. Syst..

[5] Shin J., Choi G., Woo J., Park J., Park S., Lee W., Kim S., Son M., Hwang H. (2012). MIM-type cell selector for high-density and low-power cross-point memory application. Microelectron. Eng..

[6] Permiakova O.O., Rogozhin A.E., Miakonkikh A.V., Smirnova E.A., Rudenko K.V. (2023). Transition between resistive switching modes in asymmetric HfO_2_-based structures. Microelectron. Eng..

[7] Lanza M., Waser R., Ielmini D., Yang J.J., Goux L., Suñe J., Kenyon A.J., Mehonic A., Spiga S., Rana V. (2021). Standards for the Characterization of Endurance in Resistive Switching Devices. ACS Nano.

[8] Zhang Y., Wang Z., Zhu J., Yang Y., Rao M., Song W., Zhuo Y., Zhang X., Cui M., Shen L. (2020). Brain-inspired computing with memristors: Challenges in devices, circuits, and systems. Appl. Phys. Rev..

[9] Zhang Y., Mao G.Q., Zhao X., Li Y., Zhang M., Wu Z., Wu W., Sun H., Guo Y., Wang L. (2021). Evolution of the conductive filament system in HfO_2_-based memristors observed by direct atomic-scale imaging. Nat. Commun..

[10] Vinuesa G., García H., Poblador S., González M.B., Campabadal F., Castán H., Dueñas S. (2024). Impact of the temperature on the conductive filament morphology in HfO_2_-based RRAM. Mater. Lett..

[11] Isaev A., Permyakova O., Rogozhin A. (2023). Mechanisms of conductive filament formation in hafnium oxide multilayer structures. Thin Solid Film..

[12] Padovani A., Torraca P.L., Strand J., Shluger A., Milo V., Larcher L. Towards a Universal Model of Dielectric Breakdown. Proceedings of the 2023 IEEE International Reliability Physics Symposium (IRPS).

[13] Petzold S., Zintler A., Eilhardt R., Piros E., Kaiser N., Sharath S.U., Vogel T., Major M., McKenna K.P., MolinaLuna L. (2019). Forming-Free Grain Boundary Engineered Hafnium Oxide Resistive Random Access Memory Devices. Adv. Electron. Mater..

[14] Pan J., He H., Dan Y., Lin Y., Yang S., Li M., Li T. (2023). HfO2-Based RRAM with In Situ Conductive Channels Induced by Nanoparticles to Improve Stability. ACS Appl. Electron. Mater..

[15] Sharath S.U., Bertaud T., Kurian J., Hildebrandt E., Walczyk C., Calka P., Zaumseil P., Sowinska M., Walczyk D., Gloskovskii A. (2014). Towards forming-free resistive switching in oxygen engineered HfO2—x. Appl. Phys. Lett..

[16] He W., Sun H., Zhou Y., Lu K., Xue K., Miao X. (2017). Customized binary and multi-level HfO2—x-based memristors tuned by oxidation conditions. Sci. Rep..

[17] Hildebrandt E., Kurian J., Müller M.M., Schroeder T., Kleebe H.J., Alff L. (2011). Controlled oxygen vacancy induced p-type conductivity in HfO2—x thin films. Appl. Phys. Lett..

[18] Chand U., Huang C.Y., Jieng J.H., Jang W.Y., Lin C.H., Tseng T.Y. (2015). Suppression of endurance degradation by utilizing oxygen plasma treatment in HfO_2_ resistive switching memory. Appl. Phys. Lett..

[19] Zhao X., Wang Z., Li W., Sun S., Xu H., Zhou P., Xu J., Lin Y., Liu Y. (2020). Photoassisted Electroforming Method for Reliable LowPower Organic–Inorganic Perovskite Memristors. Adv. Funct. Mater..

[20] Hsieh E.R., Chen K.T., Chen P.Y., Wong S.S., Chung S.S. (2021). A FORMing-Free HfO2-/HfON-Based Resistive-Gate Metal–Oxide–Semiconductor Field-Effect-Transistor (RG-MOSFET) Nonvolatile Memory With 3-Bit-Per-Cell Storage Capability. IEEE Trans. Electron Devices.

[21] Tian Q., Zhao X., Zhang X., Lin H., Wang D., Xing G., Wang Z., Lin Y., Xu H., Liu Y. (2021). Thermal-assisted electroforming enables performance improvement by suppressing the overshoot current in amorphous carbon-based electrochemical metallization memory. Appl. Phys. Lett..

[22] Ku B., Abbas Y., Sokolov A.S., Choi C. (2018). Interface engineering of ALD HfO_2_-based RRAM with Ar plasma treatment for reliable and uniform switching behaviors. J. Alloy. Compd..

[23] Sokolov A.S., Jeon Y.R., Ku B., Choi C. (2020). Ar ion plasma surface modification on the heterostructured TaO_X_/InGaZnO thin films for flexible memristor synapse. J. Alloy. Compd..

[24] Zhao L., Ng W.H., Knights A.P., Stevanovic D.V., Mannion D.J., Mehonic A., Kenyon A.J. (2022). Engineering Silicon Oxide by Argon Ion Implantation for High Performance Resistance Switching. Front. Mater..

[25] Mikhaylov A.N., Belov A.I., Korolev D.S., Gerasimova S.A., Antonov I.N., Okulich E.V., Shuiskiy R.A., Tetelbaum D.I. (2019). Effect of ion irradiation on resistive switching in metal-oxide memristive nanostructures. J. Phys. Conf. Ser..

[26] Zhang H., Liu L., Gao B., Qiu Y., Liu X., Lu J., Han R., Kang J., Yu B. (2011). Gd-doping effect on performance of HfO_2_ based resistive switching memory devices using implantation approach. Appl. Phys. Lett..

[27] Liu Q., Long S., Wang W., Zuo Q., Zhang S., Chen J., Liu M. (2009). Improvement of Resistive Switching Properties in ZrO_2_-Based ReRAM With Implanted Ti Ions. IEEE Electron Device Lett..

[28] Wu E.Y. (2019). Facts and Myths of Dielectric Breakdown Processes—Part I: Statistics, Experimental, and Physical Acceleration Models. IEEE Trans. Electron Devices.

[29] Suñé J., Placencia I., Barniol N., Farrés E., Martín F., Aymerich X. (1990). On the breakdown statistics of very thin SiO_2_ films. Thin Solid Film..

[30] Strand J., La Torraca P., Padovani A., Larcher L., Shluger A.L. (2022). Dielectric breakdown in HfO_2_ dielectrics: Using multiscale modeling to identify the critical physical processes involved in oxide degradation. J. Appl. Phys..

[31] Kerber A., Pantisano L., Veloso A., Groeseneken G., Kerber M. (2007). Reliability screening of high-k dielectrics based on voltage ramp stress. Microelectron. Reliab..

[32] Sassine G., Cagli C., Nodin J.F., Molas G., Nowak E. (2018). Novel Computing Method for Short Programming Time and Low Energy Consumption in HfO_2_ Based RRAM Arrays. IEEE J. Electron Devices Soc..

[33] Wu E., Ando T., Li B., Southwick R., Stathis J. (2020). Fundamental roles of extreme-value distributions in dielectric breakdown and memory applications (minimum-value versus maximum-value statistics). Jpn. J. Appl. Phys..

[34] Ziegler J.F., Ziegler M., Biersack J. (2010). SRIM – The stopping and range of ions in matter (2010). Nucl. Instrum. Methods Phys. Res. Sect. B Beam Interact. Mater. Atoms..

[35] Karwal S., Verheijen M.A., Arts K., Faraz T., Kessels W., Creatore M. (2020). Plasma-Assisted ALD of Highly Conductive HfNx: On the Effect of Energetic Ions on Film Microstructure. Plasma Chem. Plasma Process..

[36] Zhang H., Ju X., Zhou Y., Gu C., Pan J., Ang D.S. (2019). Realization of Self-Compliance Resistive Switching Memory via Tailoring Interfacial Oxygen. ACS Appl. Mater. Interfaces.

