# Supplementary material for: Setting Plasma Immersion Ion Implantation of Ar+ Parameters towards Electroforming-Free and Self-Compliance HfO2-Based Memristive Structures
Source: Nanomaterials (Basel). 2024 May 9;14(10):831. doi: 10.3390/nano14100831 (PMC11123830; doi:10.3390/nano14100831)
Supplement: Supplementary file 1 [file nanomaterials-14-00831-s001.zip › nanomaterials-2926125-supplementary.pdf]

Supplementary Materials: Setting plasma immersion ion implantation of Ar^+ parameters towards electroforming-free and self-compliance HfO_2-based memristive structures

Olga Permiakova *, Sergey Pankratov, Alexandr Isaev, Andrew Miakonkikh, Yuri Chesnokov, Andrey Lomov and Alexander Rogozhin *

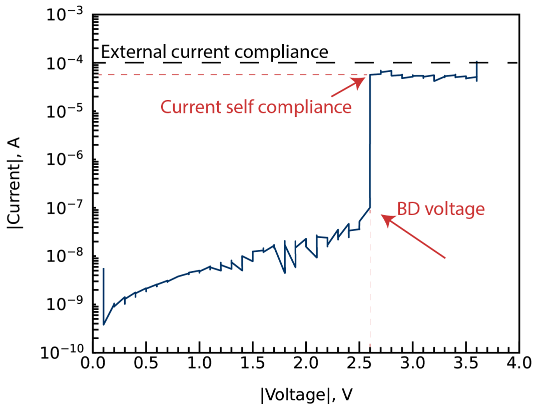

Figure S1. Example of electroforming process of $Pt/HfO_2/HfO_XN_Y/TaN$ after plasma immersion ion implantation of Ar^+ with energy 2 keV and fluence $7.0 \cdot 10^{15}$ cm^{-2}.

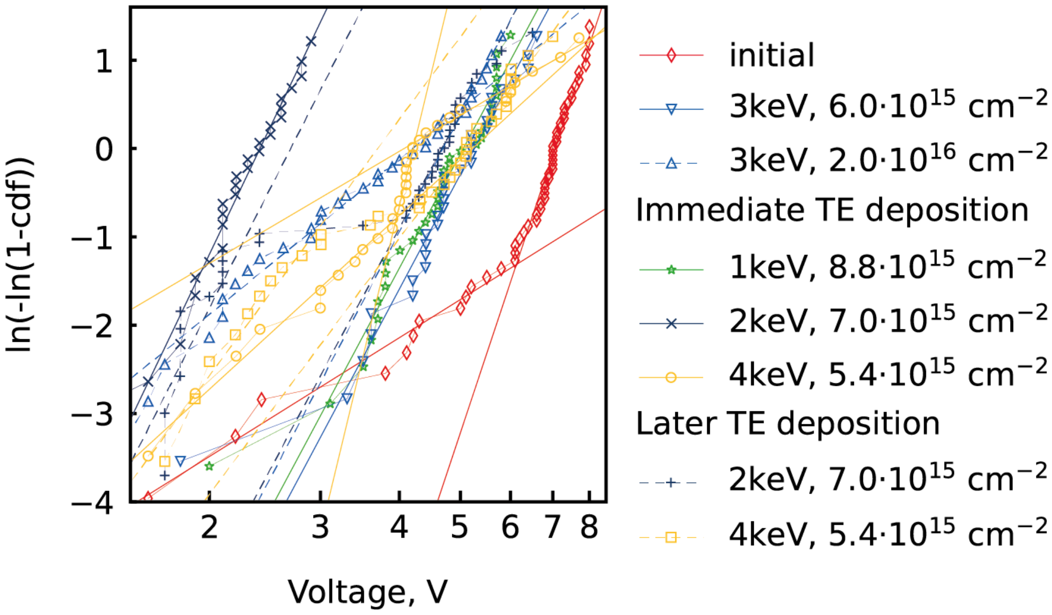

Figure S2. Fitted Weibull distributions of breakdown voltages for $Pt/HfO_2/HfO_XN_Y/TaN$ structures subjected to plasma immersion ion implantation of Ar^+.

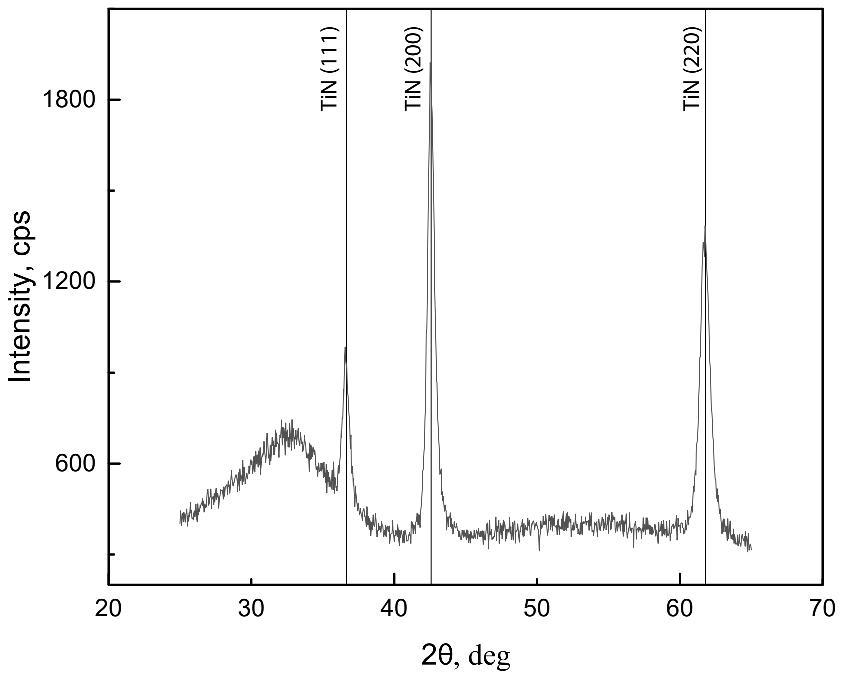

Figure S3. GIXRD spectra of the $HfO_2/HfO_XN_Y/TaN$ structure without implantation.

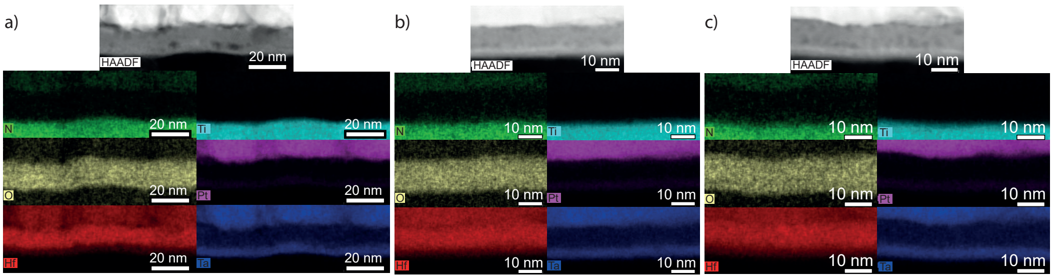

Figure S4. Cross-sectional HAADF-STEM image of the $Pt/HfO_2/HfO_XN_Y/Ta$ structures and corresponding EDS mapping of N, Ti, O, Pt, Hf and Ta without deconvolution. a) Cross-sectional HAADF-STEM image of the reference structure. b) Cross-sectional HAADF-STEM image of the structure after Ar^+ plasma immersion ion implantation with the energy of 2 keV and fluence of $7.0 \cdot 10^{15}$ cm^{-2}. c) Cross-sectional HAADF-STEM image of the structure after Ar^+ plasma immersion ion implantation with the energy of 4 keV and fluence of $5.4 \cdot 10^{15}$ cm^{-2}

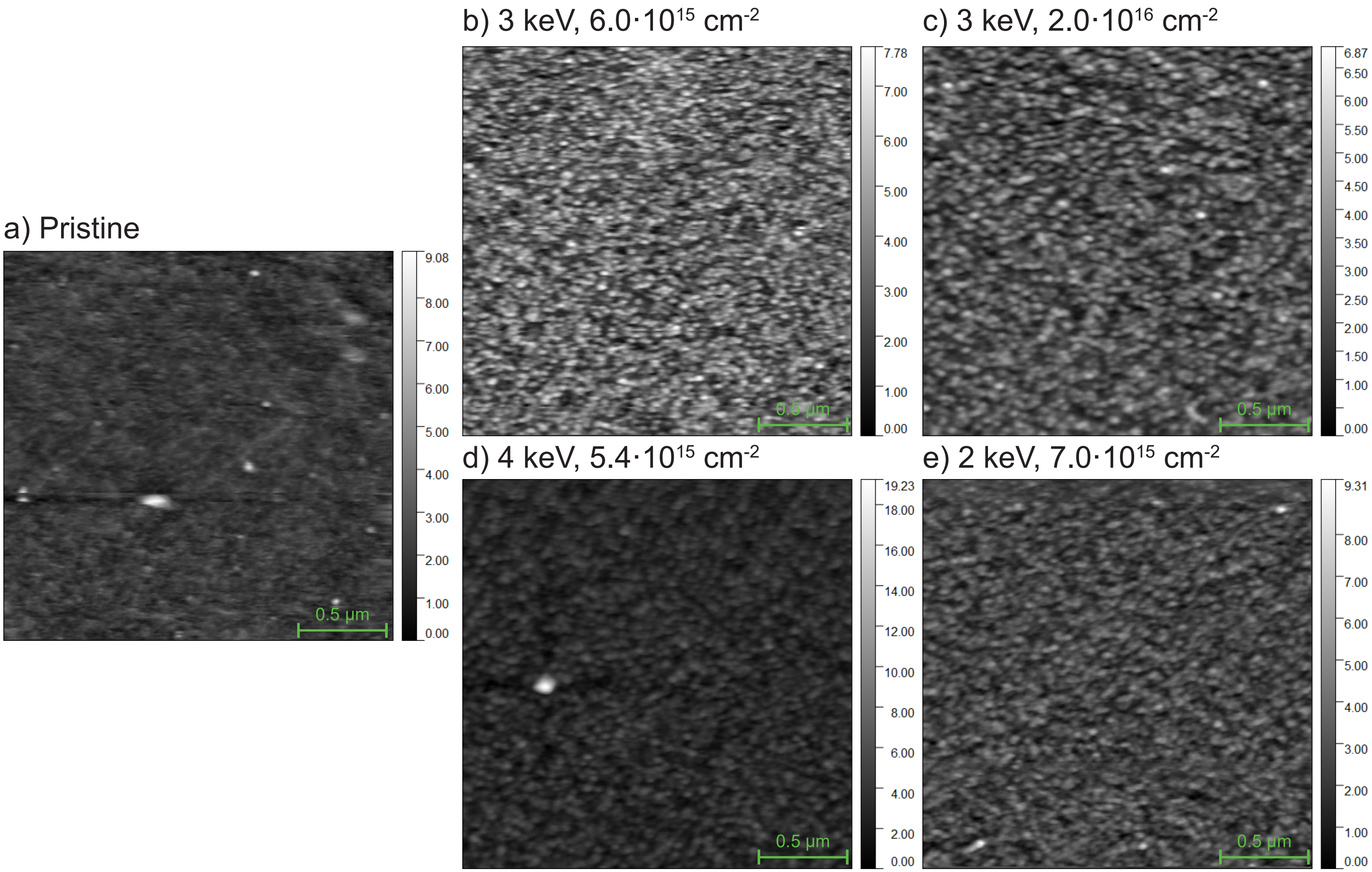

Figure S5. Images used to estimate surface roughness measured via AFM. The images were processed with a Fourier filter.

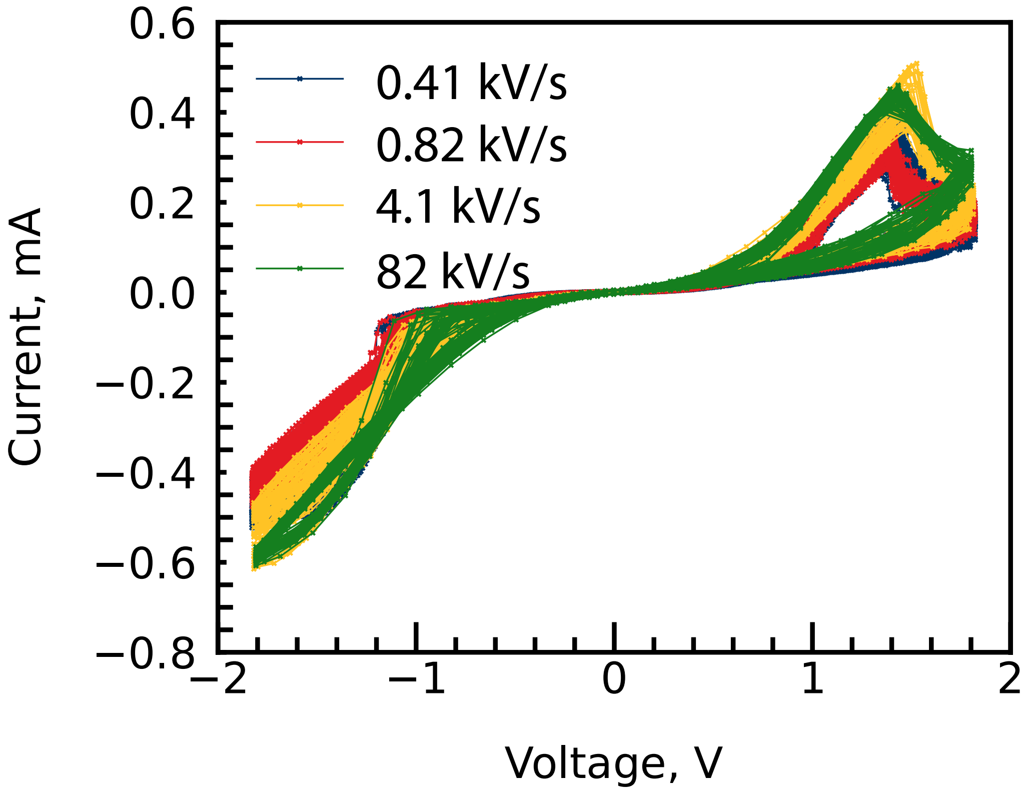

Figure S6. I-V characteristics of Pt/HfO_2/HfO_XN_Y/TaN structures subjected to plasma immersion ion implantation of Ar^+ with energy of 2 keV and fluence of $7 \cdot 10^{15}$ cm^{-2}.

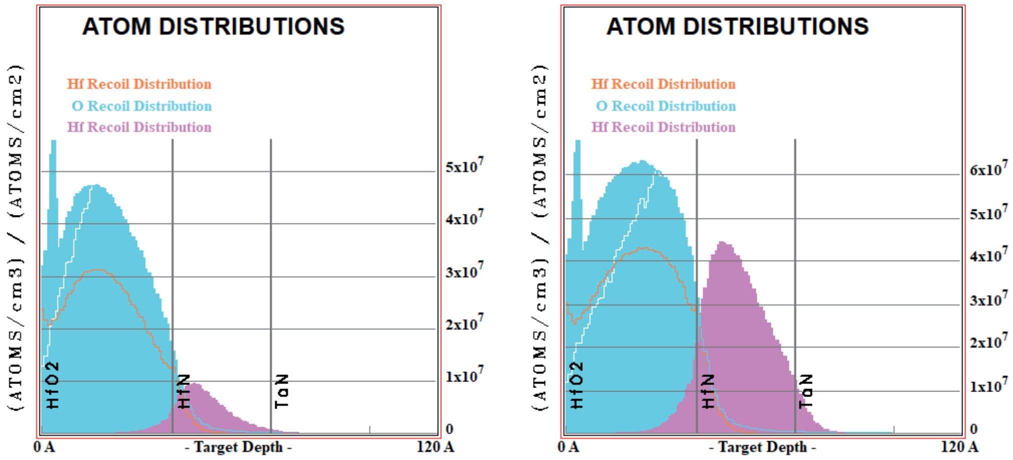

Figure S7. SRIM simulation of Hf atom displacements induced by Ar^+ plasma immersion ion implantation in HfO_2/HfO_XN_Y/TaN structure with energies of 2 keV (a) and of 4 keV (b).
